# Use of and Steering to Pharmacies Owned by Insurers and Pharmacy Benefit Managers in Medicare

**DOI:** 10.1001/jamahealthforum.2024.4874

**Published:** 2025-01-10

**Authors:** Pragya Kakani, Swayami Navangul, Christie Lee Luo, Kayla N. Tormohlen, Genevieve P. Kanter, Mary Beth Landrum, Nancy L. Keating, Amelia M. Bond

**Affiliations:** 1 Department of Population Health Sciences, Weill Cornell Medical College, New York, New York; 2 Department of Health Policy and Management, Sol Price School of Public Policy, University of Southern California, Los Angeles; 3 Leonard D. Schaeffer Center for Health Policy and Economics, University of Southern California, Los Angeles; 4 Department of Health Care Policy, Harvard Medical School, Boston, Massachusetts; 5 Division of General Internal Medicine, Brigham and Women’s Hospital, Boston, Massachusetts

## Abstract

**Importance:**

The prevalence of pharmacies owned by integrated insurers and pharmacy benefit managers (PBMs), or insurer-PBMs, is of growing regulatory concern. However, little is known about the role of these pharmacies in Medicare, in which pharmacy network protections may influence market dynamics.

**Objective:**

To evaluate the prevalence of insurer-PBM–owned pharmacies and the extent to which insurer-PBMs steer patients to pharmacies they own in Medicare.

**Design, Setting, and Participants:**

This cross-sectional study used Medicare Part D claims data on prescription fills for a 20% random sample of US beneficiaries enrolled from January 1 through December 31, 2021. Data were analyzed from March to November 2024.

**Exposures:**

Prescription fills.

**Main Outcomes and Measures:**

The main outcome was the share of spending filled by insurer-PBM–owned pharmacies overall, by pharmacy type (specialty and nonspecialty), and by drug class. For the top 100 specialty and nonspecialty molecules by claim volume, 2 quantities were identified for 4 major insurer-PBMs (Cigna, CVS, Humana, and UnitedHealth Group): share of the index firm’s insurer claims filled by its owned pharmacies and share of other firms’ insurer claims filled by the index firm’s owned pharmacies. Differences between these quantities were assessed to evaluate the degree to which insurer-PBMs steered patients to their own pharmacies.

**Results:**

Among 10 455 726 patients (54.8% women; mean [SD] age, 71.8 [10.7] years), 34.1% of all pharmacy and 37.1% of specialty pharmacy spending occurred through Cigna, CVS, Humana, or UnitedHealth Group pharmacies. Among specialty molecules, market shares varied by drug class (antivirals: 18.5%; antipsychotics: 29.5%; cancer: 32.5%; disease-modifying antirheumatic drugs: 41.1%; multiple sclerosis: 64.8%; pulmonary arterial hypertension and idiopathic pulmonary fibrosis: 89.7%). Across molecule-firm combinations, a 19.8 (95% CI, 18.0-21.6)–percentage point and 13.9 (95% CI, 13.1-14.7)–percentage point greater share of claims were filled at insurer-PBM–owned pharmacies than would be expected without steering for specialty and nonspecialty categories, respectively.

**Conclusions and Relevance:**

This cross-sectional study found that insurer-PBM firms represented an important portion of the Medicare Part D market, especially for certain drug classes, and that insurer-PBM firms steered patients to their own pharmacies, despite certain pharmacy network protections in Medicare. These findings underscore the need to understand the impacts of insurer-PBM and pharmacy integration on medication access and costs for Medicare patients.

## Key Points

**Question:**

In Medicare Part D, how prevalent are pharmacies owned by insurers and pharmacy benefit managers (PBMs), and to what extent do insurer-PBM firms steer patients to pharmacies they own?

**Findings:**

In this cross-sectional study of Medicare Part D claims data for 10 455 726 patients, 34.1% of all pharmacy and 37.1% of specialty pharmacy spending occurred through Cigna-, CVS-, Humana-, or UnitedHealth Group–owned pharmacies in 2021, with variation across drug classes. Each firm steered plan enrollees to their own pharmacies.

**Meaning:**

The findings suggest that insurer-PBM–owned pharmacies represent an important portion of the Medicare market partly because firms steer patients to their own pharmacies, highlighting the need to understand the implications of insurer-PBM and pharmacy integration.

## Teaser

This cross-sectional study uses Medicare Part D claims data to evaluate the share of pharmacies owned by insurers and pharmacy benefit managers (PBMs) and the extent to which insurer-PBM firms steer patients to pharmacies they own.

## Introduction

There is growing regulatory concern regarding the prevalence of pharmacies owned by insurers and pharmacy benefit managers (PBMs), which are organizations that negotiate pharmacy and manufacturer contracts and have increasingly integrated with insurers to form insurer-PBM firms.^1^ Pharmacies owned by insurer-PBMs have significant market power in certain segments, with industry reports suggesting that pharmacies owned by 3 insurer-PBM firms account for over two-thirds of the specialty and mail-order markets nationally.^1,2^ Moreover, insurer-PBMs face incentives to steer patients to pharmacies they own. This can be done by leaving other pharmacies out of network,^3^ including their own pharmacies in preferred networks with lower out-of-pocket costs,^4^ and advertising to plan enrollees.^5^ Steering may improve welfare if it helps patients access medicines or if these pharmacies offer more cost-effective services. However, steering may impede rival pharmacies’ ability to compete, harming patients who may prefer these pharmacies.^1,5^ Insurer-PBMs may also overpay their own pharmacies to pass costs to plan sponsors or depress reported profits.^1,6,7,8^

The prevalence and behavior of insurer-PBM–owned pharmacies may differ by market, and market-specific insights are needed to inform regulators. For example, in Medicare Part D, Any Willing Pharmacy rules prevent plans from leaving pharmacies out of network if they meet plan-defined, standard criteria that are reasonable and relevant.^9^ By removing one mechanism by which insurer-PBMs can steer patients to their own pharmacies, this policy may reduce use of these pharmacies. Nonetheless, the continued ability to use preferred networks and marketing may still permit steering. Therefore, we quantified the use of and steering to insurer-PBM–owned pharmacies in Medicare Part D.

## Methods

This cross-sectional study used Medicare Part D claims data (20% sample) from Medicare Advantage prescription drug and stand-alone prescription drug plans from January 1 to December 31, 2021, along with a May 2021 snapshot of the National Council for Prescription Drug Program pharmacy directory. We excluded Medicare beneficiaries from outside the 50 US states and the District of Columbia. For 2.2% of claims with zero spending, spending was inferred using the median spending among claims with nonzero spending for the same National Drug Code (eAppendix 1 in Supplement 1). This study followed the Strengthening the Reporting of Observational Studies in Epidemiology (STROBE) reporting guideline and was approved by the Weill Cornell institutional review board, with a waiver of informed consent because the data were collected previously for billing purposes and do not include patient identifiers.

Following methods from prior work,^10,11^ we identified pharmacies owned by conglomerate firms that also owned the 4 largest PBMs and Part D insurance plans (ie, insurer-PBM firms): Cigna, CVS, Humana, and UnitedHealth Group (UHG).^12^ We identified specialty drug molecules costing more than $10 000 per patient-year^11^ and defined drug classes. We stratified pharmacies into specialty and nonspecialty pharmacies, defining specialty pharmacies as those in which more than 25% of spending was for specialty drugs. Details on identifying insurer-PBM–owned pharmacies and plans are given in eAppendix 2; drug classes, in eAppendix 3; pharmacy types, in eAppendix 4 and eFigure 1; and firm characteristics, in eTables 1 and 2 in Supplement 1.

### Statistical Analysis

We first sought to describe the overall use of insurer-PBM–owned pharmacies. Specifically, we reported the share of spending at insurer-PBM–owned pharmacies overall and by pharmacy type, specialty and nonspecialty drug categories, and drug class within each category.

We then tested for steering (ie, preferential use of owned pharmacies) of enrollees in Cigna, CVS, Humana, and UHG insurance plans. For the top 100 specialty and top 100 nonspecialty molecules by claim volume, we identified 2 quantities for each index insurer-PBM: (1) share of the index firm’s insurer claims filled by its owned pharmacies and (2) share of other firms’ insurer claims filled by the index firm’s owned pharmacies. For example, for CVS and atorvastatin, we identified (1) the share of atorvastatin claims in CVS Part D plans filled by CVS-owned pharmacies and (2) the share of atorvastatin claims in non-CVS Part D plans filled by CVS-owned pharmacies. If firms did not steer and used their own pharmacies at the same rate as other insurers, these quantities would be equivalent. Thus, we tested for differences using paired-sample *t* tests. To address differences due to geographic co-location rather than steering, we also constructed geography-adjusted measures by estimating county-level measures and aggregating nationally (eAppendix 5 in Supplement 1). eAppendix 6 in Supplement 1 shows the share of all claims that each insurer-PBM–owned pharmacy represented of their own vs other plans as an alternative measure of steering. Data were analyzed from March to November 2024 using Stata, version 14.0 (StataCorp LLC).

## Results

**Figure 1.**
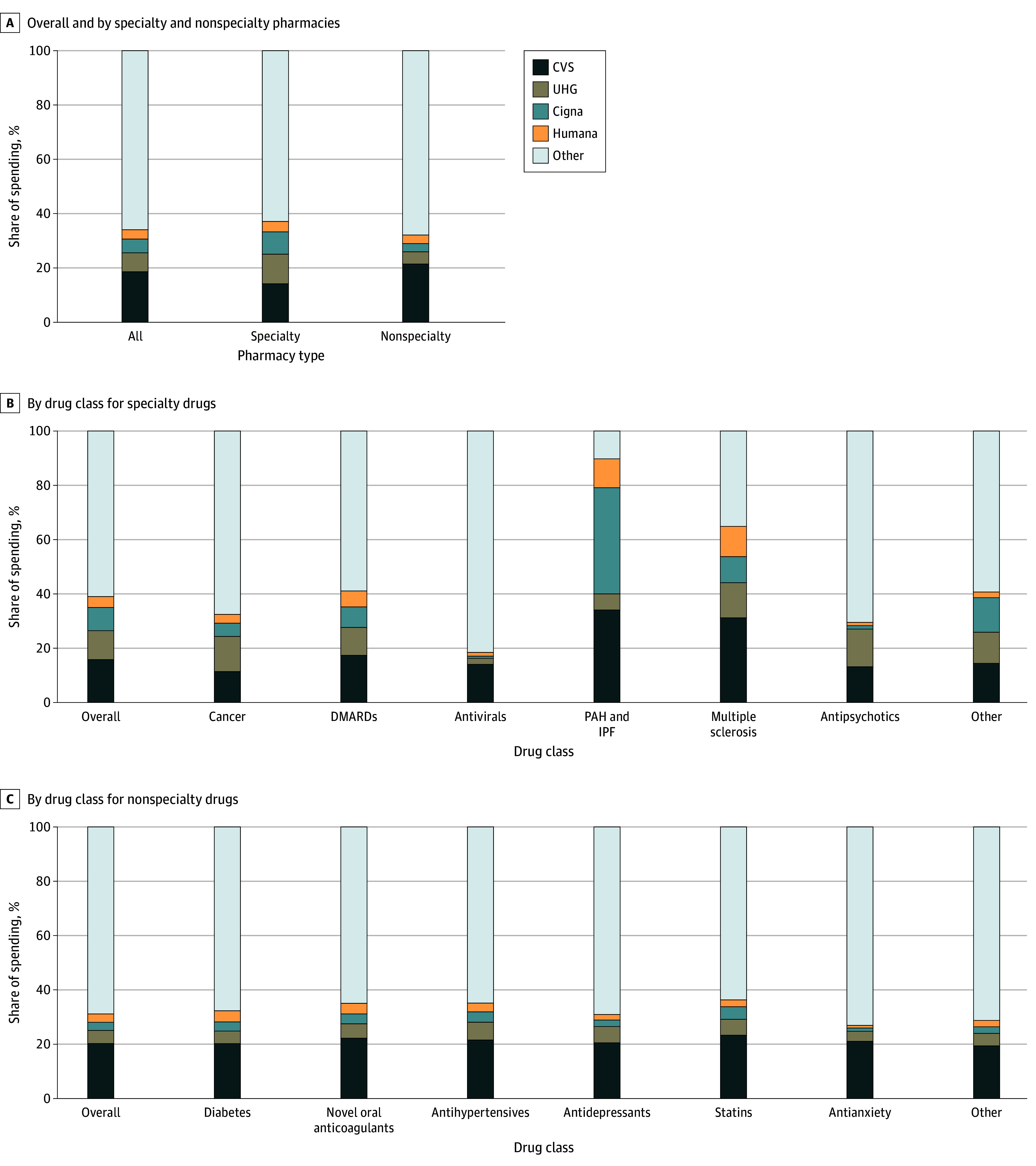
Medicare Part D Spending Filled Through Cigna, CVS, Humana, or UnitedHealth Group (UHG) Pharmacies in 2021 eAppendix 3 in Supplement 1 gives details on methods for identifying drug classes. DMARDs indicates disease-modifying anti-rheumatic drugs; IPF, idiopathic pulmonary fibrosis; PAH, pulmonary arterial hypertension.

We studied 10 455 726 patients (45.2% men; 54.8% women; mean [SD] age, 71.8 [10.7] years) and found that 34.1% of all pharmacy, 37.1% of specialty pharmacy, and 32.1% of nonspecialty pharmacy spending occurred through pharmacies owned by Cigna, CVS, Humana, or UHG (Figure 1). Among specialty molecules, there was considerable heterogeneity by class (overall: 39.0%; antivirals: 18.5%; antipsychotics: 29.5%; cancer: 32.5%; disease-modifying antirheumatic drugs: 41.1%; multiple sclerosis: 64.8%; pulmonary arterial hypertension and idiopathic pulmonary fibrosis: 89.7%). Nonspecialty classes were less heterogeneous (eTable 3 in Supplement 1).

**Figure 2.**
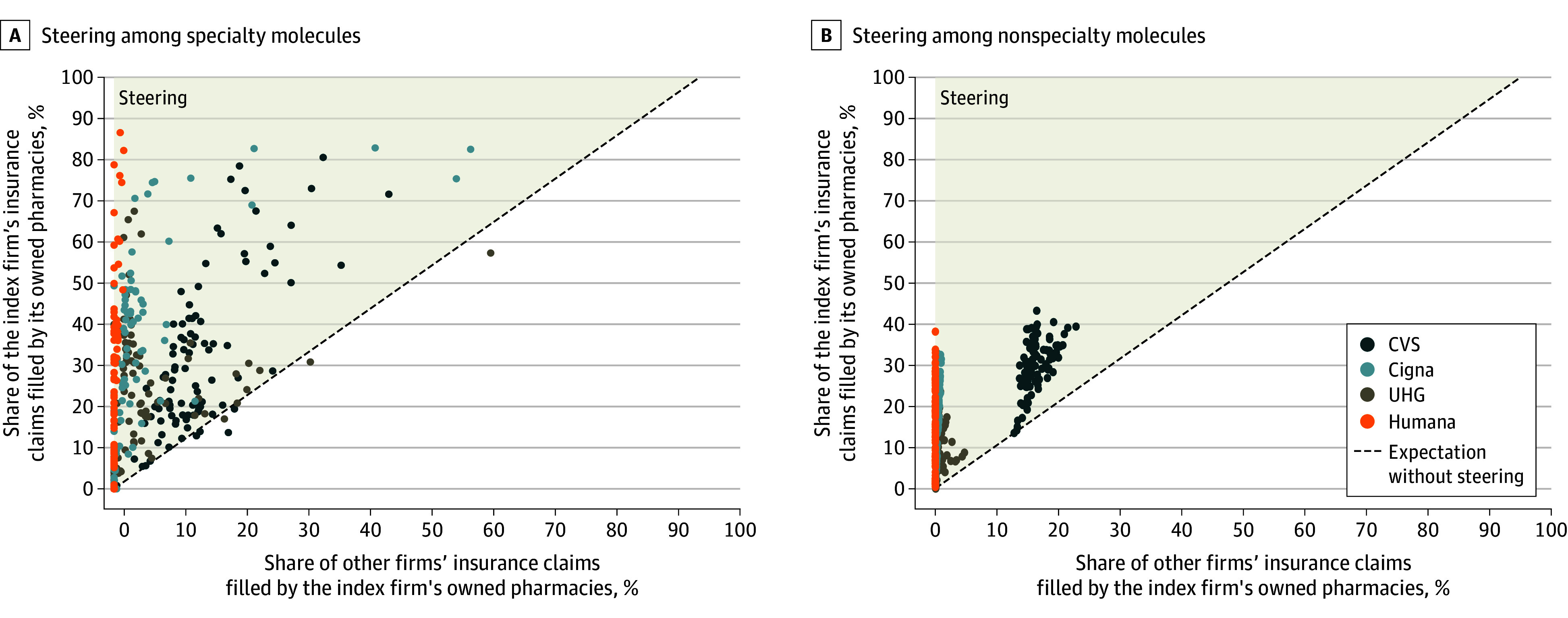
Steering of Claims for the Top 100 Specialty and Nonspecialty Molecules to Insurer-Pharmacy Benefit Manager (PBM)–Owned Pharmacies in 2021 Insurer-molecule pairs for which either coordinate was based on data from fewer than 11 patients were censored to 0 to satisfy cell-size suppression rules in Centers for Medicare & Medicaid data use agreements. The highlighted area indicates that the share of the index firm’s insurance claims filled by its owned pharmacies was greater than the expectation without steering. UHG indicates UnitedHealth Group.

We also found evidence of steering (Figure 2). Among molecule-firm pairs, 91.0% of specialty and 99.8% of nonspecialty observations had more claims filled at insurer-owned pharmacies than would be expected without steering. Moreover, for each insurer-PBM and drug category, a significantly greater share of insurer-PBM insurance claims were filled at owned pharmacies than would be expected without steering (Table). For specialty drugs, this difference was 19.8 (95% CI, 18.0-21.6) percentage points (pp) overall, 13.9 (95% CI, 11.5-16.3) pp for CVS, 11.9 (95% CI, 9.7-14.2) pp for UHG, 21.7 (95% CI, 18.2-25.3) pp for Cigna, and 18.9 (95% CI, 15.3-22.4) pp for Humana. For nonspecialty drugs, this difference was 13.9 (95% CI, 13.1-14.7) pp overall, 11.6 (95% CI, 10.7-12.5) pp for CVS, 8.5 (95% CI, 7.4-9.6) pp for UHG, 12.9 (95% CI, 11.3-14.5) pp for Cigna, and 18.9 (95% CI, 15.3-22.4) pp for Humana. These results were robust to geographic adjustment (eTable 4 in Supplement 1) and to an alternative measure of steering based on all claims (eFigure 2 in Supplement 1).

**Table.** Estimated Steering of Claims for Top 100 Specialty and Nonspecialty Molecules to Insurer-Pharmacy Benefit Manager–Owned Pharmacies in 2021

Pharmacy	Share, median (IQR), %		Pair-wise difference, percentage points	
	Index firm’s insurer claims filled by its owned pharmacies	Other firm’s insurer claims filled by the index firm’s owned pharmacies	Median (IQR)	Mean (95% CI)
**Specialty drugs**				
CVS	24.7 (17.7-40.4)	12.2 (9.0-15.9)	12.8 (5.2-26.3)	16.8 (13.9-19.7)
UHG	19.7 (4.9-31.4)	2.1 (0.6-4.4)	9.4 (3.6-27.6)	16.0 (12.8-19.1)
Cigna	25.4 (4.9-44.6)	1.7 (0.2-3.3)	23.2 (4.7-40.5)	24.0 (20.0-27.9)
Humana	16.6 (5.8-36.9)	0.1 (0.0-0.4)	16.6 (5.7-36.3)	22.5 (18.2-26.7)
All firms	21.1 (6.7-37.7)	1.7 (0.2-8.5)	14.9 (4.7-31.4)	19.8 (18.0-21.6)
**Nonspecialty drugs**				
CVS	30.4 (26.4-34.4)	15.7 (14.9-17.4)	13.7 (11.1-17.0)	13.8 (12.8-14.9)
UHG	11.8 (6.3-17.3)	0.4 (0.3-0.7)	11.3 (4.4-16.6)	11.0 (9.7-12.4)
Cigna	15.4 (5.7-21.0)	0.4 (0.2-0.6)	15.0 (5.6-20.5)	14.1 (12.4-15.8)
Humana	17.7 (9.0-24.4)	0 (0.0-0.0)	17.7 (9.0-24.3)	16.8 (14.9-18.6)
All firms	18.1 (8.8-26.6)	0.4 (0.0-8.8)	14.0 (7.4-19.4)	13.9 (13.1-14.7)

Abbreviation: UHG, UnitedHealth Group.

## Discussion

This cross-sectional study found that 34.1% of all pharmacy and 37.1% of specialty pharmacy spending in Medicare Part D occurred through pharmacies owned by Cigna, CVS, Humana, and UHG. While substantial, these estimates are considerably lower than national estimates, potentially due to Medicare’s pharmacy network protections.^1,2^ Nonetheless, in select classes, over 60% of spending was filled through insurer-PBM–owned pharmacies, which may reflect limited pharmacy competition and restricted distribution networks for certain products that exclude most non–insurer-PBM pharmacies. Meanwhile, for cancer and antiviral classes, insurer-PBM–owned pharmacies were less prominent, potentially owing to the rise of practice and health system pharmacies in these classes partly related to 340B Drug Discount Program incentives.^11^

All 4 leading insurer-PBM firms meaningfully steered beneficiaries to their own pharmacies. This steering could have occurred because insurers included their own pharmacies in preferred networks with lower co-pays,^4^ limited standard network inclusion with criteria such as requiring licensure in all 50 states or offering poor reimbursement,^13^ or marketed to patients.^5^ Moreover, some insurer-PBM–owned pharmacies do not enter other insurers’ networks.^14^

### Limitations

This study has several limitations. First, we were unable to comprehensively compare the use of insurer-PBM–owned pharmacies across all insurance segments. Second, we did not examine the mechanisms underlying observed patterns. Third, we did not evaluate steering by PBMs to owned pharmacies in cases in which insurers and PBMs were not integrated. Fourth, we did not evaluate the welfare implications of integration between insurer-PBMs and pharmacies. Characterizing welfare will be important for future research and will depend on the impacts on patient out-of-pocket costs and access, pharmacy competition and choice, quality of care, and prices paid by plan sponsors.

## Conclusions

This cross-sectional study found that insurer-PBM firms represented an important portion of the Medicare Part D market, especially for certain drug classes, and that insurer-PBM firms steered patients to their own pharmacies, despite certain pharmacy network protections in Medicare. These findings underscore the need to understand the impacts of insurer-PBM and pharmacy integration on medication access and costs for Medicare patients.

## Notes

Supplement 1. **eAppendix 1.** Treatment of Medicare Part D claims with zero total cost **eAppendix 2.** Identifying pharmacies owned by and plans operated by CVS, UHG, Cigna, and Humana **eTable 1.** Key pharmacy brands associated with CVS, UHG, Cigna, and Humana **eAppendix 3.** Identifying specialty and non-specialty molecules and drug classes among specialty and non-specialty categories **eAppendix 4.** Identifying pharmacy types **eFigure 1.** Cumulative share of spending on non-specialty and specialty drugs, by share of pharmacy spending on specialty drugs (2021) **eTable 2.** Characteristics of pharmacies owned by CVS, UHG, Cigna, and Humana **eAppendix 5.** Construction of market share terms underlying main text Figure 2 and Table **eAppendix 6.** Supplemental analysis **eTable 3.** Share of Medicare Part D spending filled through pharmacies owned by CVS, UHG, Cigna, or Humana in 2021 **eTable 4.** Geography-adjusted estimated steering of claims for top-100 specialty and non-specialty molecules to insurer-PBMs’ owned pharmacies (2021) **eFigure 2**. Share of each insurer-PBMs’ Part D plan claims filled through its own pharmacy vs the corresponding share for all other Part D plans (2021)

Supplement 2.
Data Sharing Statement
