# Supplementary material for: Use of and Steering to Pharmacies Owned by Insurers and Pharmacy Benefit Managers in Medicare
Source: JAMA Health Forum. 2025 Jan 10;6(1):e244874. doi: 10.1001/jamahealthforum.2024.4874 (PMC11724340; doi:10.1001/jamahealthforum.2024.4874)
Supplement: Supplement 1. — eAppendix 1. Treatment of Medicare Part D claims with zero total cost eAppendix 2. Identifying pharmacies owned by and plans operated by CVS, UHG, Cigna, and Humana eTable 1. Key pharmacy brands associated with CVS, UHG, Cigna, and Humana eAppendix 3. Identifying specialty and non-specialty molecules and drug classes among specialty and non-specialty categories eAppendix 4. Identifying pharmacy types eFigure 1. Cumulative share of spending on non-specialty and specialty drugs, by share of pharmacy spending on specialty drugs (2021) eTable 2. Characteristics of pharmacies owned by CVS, UHG, Cigna, and Humana eAppendix 5. Construction of market share terms underlying main text Figure 2 and Table eAppendix 6. Supplemental analysis eTable 3. Share of Medicare Part D spending filled through pharmacies owned by CVS, UHG, Cigna, or Humana in 2021 eTable 4. Geography-adjusted estimated steering of claims for top-100 specialty and non-specialty molecules to insurer-PBMs’ owned pharmacies (2021) eFigure 2. Share of each insurer-PBMs’ Part D plan claims filled through its own pharmacy vs the corresponding share for all other Part D plans (2021) [file jamahealthforum-e244874-s001.pdf]

Supplemental Online Content

Kakani P, Navangul S, Lee Luo C et al. Use of and steering to pharmacies owned by insurers and pharmacy benefit managers in Medicare. *JAMA Health Forum.* Published online January 10, 2025. doi:10.1001/jamahealthforum.2024.4874

eAppendix 1. Treatment of Medicare Part D claims with zero total cost

eAppendix 2. Identifying pharmacies owned by and plans operated by CVS, UHG, Cigna, and Humana

eTable 1. Key pharmacy brands associated with CVS, UHG, Cigna, and Humana

eAppendix 3. Identifying specialty and non-specialty molecules and drug classes among specialty and non-specialty categories

eAppendix 4. Identifying pharmacy types

eFigure 1. Cumulative share of spending on non-specialty and specialty drugs, by share of pharmacy spending on specialty drugs (2021)

eTable 2. Characteristics of pharmacies owned by CVS, UHG, Cigna, and Humana

eAppendix 5. Construction of market share terms underlying main text Figure 2 and Table

eAppendix 6. Supplemental analysis

eTable 3. Share of Medicare Part D spending filled through pharmacies owned by CVS, UHG, Cigna, or Humana in 2021

eTable 4. Geography-adjusted estimated steering of claims for top-100 specialty and non-specialty molecules to insurer-PBMs' owned pharmacies (2021)

eFigure 2. Share of each insurer-PBMs' Part D plan claims filled through its own pharmacy versus the corresponding share for all other Part D plans (2021)

This supplemental material has been provided by the authors to give readers additional information about their work.

© 2025 Kakani P et al. *JAMA Health Forum.*

eAppendix 1. Treatment of Medicare Part D claims with zero total cost

We observed that 2.2% of all Medicare Part D claims in 2021 had $0 total cost; there were no claims that were missing total cost. In investigating claims with $0 total cost, we observed >92% of these claims were from two pharmacies owned by Humana, and >99% were for non-specialty drugs including anti-hypertensives, anti-depressants, statins, and diabetes medications. We determined that these claims were very likely to reflect true claims where patients were dispensed medications, but for which total cost was listed as zero, for multiple reasons. First, Medicare Part D claims reflect final action claims and thus are not meant to include denied claims. Second, we used Medicare Part D data from 2015 to 2022 to review the number of claims filled by these two Humana pharmacies in each year from 2015 to 2022 and the share of these claims that had $0 total costs. These two pharmacies did not have claims with $0 total costs prior to 2021, but most claims at these pharmacies had $0 total costs in 2021 and 2022. We determined that if claims with $0 total cost reflected truly dispensed medications, this would be most consistent with historical trends in the number of claims filled at these pharmacies. Finally, in correspondence with a former senior Humana executive, we learned that Humana covered many low-cost generic drugs with $0 cost sharing for many members. Listing the total cost as $0 may have ensured that no patients had any cost sharing. Because Humana pharmacies essentially only serve Humana plan enrollees and because these are low-cost drugs, the downside to Humana of listing total cost as $0 was also limited, as these pharmacy payments (which reflect a bill from Humana pharmacies to Humana plans) only reflect a within-firm transfer. For these reasons, claims with $0 total cost were included in our analysis. However, spending was estimated for claims with $0 total cost based on the median spending among claims with non-zero spending for the same National Drug Code.

eAppendix 2. Identifying pharmacies and plans owned by CVS, UHG, Cigna, and Humana

We identified pharmacies and plans that were owned four insurer-PBM firms: CVS Health Corporation (aka CVS), UnitedHealth Group Incorporated (aka UHG), Cigna Corporation (aka Cigna), and Humana Inc. (aka Humana). These firms are referred to as insurer-PBM firms as each firm owned both insurance plans and a pharmacy benefit manager in 2021,[i] and thus the pharmacies that the parent firms owned are referred to as insurer-PBM owned pharmacies. Importantly, the insurance plans and pharmacies are often operated by different subsidiaries of the parent firm. For example, CVS operates Part D plans under its SilverScript Insurance Company subsidiary and pharmacies under numerous other subsidiaries. In this study, we considered pharmacies and plans to be owned by same firm if they were owned by the same parent firm, regardless of subsidiary structure.

Insurer-PBM owned pharmacies were identified through a multi-step process. For each insurer-PBM firm in our sample, namely UHG, CVS, Cigna, and Humana, we first constructed a database of legacy pharmacy brands (e.g., OptumRx for UHG) and pharmacy acquisitions. This database was constructed from multiple sources including news reports, industry reports, Standard & Poor Capital IQ, Pitchbook, Securities and Exchange Commission filings, and government reports; because these firms are large public companies, most brands and transactions are widely reported. We required validation of each transaction or brand by two sources. We reviewed our brand and transaction list with former industry executives to ensure accuracy and virtual completeness.

We then used the 2021 National Council on Prescription Drugs Program (NCPDP) pharmacy directory to identify pharmacies, identified by an NCPDP identifier, that corresponded to a legacy pharmacy brand or a pharmacy acquired by the insurer-PBM firm prior to 2021. The NCPDP data included multiple business names, a parent organization, and an email address, including website domain. First, we classified any pharmacies that listed Optum, CVS, or Accredo (a subsidiary of Cigna) as the parent organization as belonging to UHG, CVS, and Cigna, respectively. Because this field did not capture Humana and may have excluded certain subsidiaries, we identified additional pharmacies based on fuzzy name matching between business names and known brands or subsidiaries of each insurer. We then manually reviewed all the website domains for identified pharmacies and specified website domains for each insurer that were confirmed to be associated with the insurer. We identified any additional pharmacies associated with those website domains. We then manually reviewed any pharmacies that were not identified by the parent organization field and did not have a valid website domain and excluded any erroneous pharmacies identified. Finally, we validated the virtual completeness of our list by manually reviewing the top 200 pharmacies by total, non-specialty, or specialty spending to assure appropriate classification of these pharmacies. These top pharmacies accounted for 33% of total spending and 60% of specialty drug spending. This approach is similar to that used in prior work to characterize pharmacy ownership in other settings.[1,2] The pharmacy brands captured and number of pharmacies by Insurer-PBM are presented below in eTable 1.

[i] UHG owned Optum, CVS owned Caremark, Cigna owned Express Scripts, and Humana owned Humana Pharmacy Solutions

© 2025 Kakani P et al. *JAMA Health Forum*.

eTable 1. Key pharmacy brands associated with CVS, UHG, Cigna, and Humana

Insurer-PBM	Key pharmacy brands operating in 2021	Number of pharmacies
CVS*	CVS, Caremark, Coram, Procare, Advanced Care Scripts, Omnicare, Geneva Woods, Navarro Discount Pharmacy, Long Drug Stores, EncompassRx	10,227
UHG	Optum, Avella, Genoa Healthcare-QoL Meds, Diplomat (including subsidiaries BioRx, Focus Rx and American Home Care Foundation), Divvydose	669
Cigna	Accredo, Cigna, Express Scripts (ESI), Freedom Fertility	60
Humana	Humana**, Enclara Pharmacia	49

Note: *CVS subsidiaries are very well-captured under the CVS parent organization field; only four additional pharmacies were not identified via this field; **Humana rebranded its pharmacies to the Centerwell brand name in 2021, but this rebrand was not reflected in the 2021 NCPDP data. Abbreviations include UnitedHealth Group (UHG).

Part D plans operated by each firm were identified using the parent organization field in the Medicare Plan Characteristics File from 2021. Our data showed that in December 2021, the share of Part D enrollees by insurer was as follows: UHG (22%), Humana (17%), CVS Health (16%), and Cigna (8%). Estimated 2021 market shares for each firm's insurance plans closely matched public estimates,[3] validating this approach.

eAppendix 3. Identifying specialty and non-specialty molecules and drug classes among specialty and non-specialty categories

Molecules were identified using the generic name field in Part D claims. However, because the same generic name can, in some cases, be written differently on different claims (e.g. imatinib and imatinib mesylate), steps were taken to standardize molecule names. First, National Drug Codes (NDCs) were assigned to a molecule name and brand name based on the molecule and brand name appearing on the plurality of Part D claims across multiple years (2010-2021). Second, several terms generally referring to non-active moieties[ii] were removed from molecule names, when not in the first position, to isolate active moieties. Third, any NDCs with the same brand name were assigned a single molecule name. Finally, in highlighted classes of interest, molecule names were manually reviewed. Molecules were then categorized as specialty and non-specialty based on the empirically observed average spending per patient among all patients with Medicare Part D claims in our sample in 2021. The $10,000 per patient-per year threshold for defining specialty molecules is consistent with recent work.[1]

We used NDCs to stratify claims for specialty and non-specialty molecules into classes, for analysis presented in Figure 1. NDCs were first assigned Therapeutic Chemical (ATC) codes using the RxNorm database from the National Library of Medicine.

Claims for specialty molecules were stratified into claims for cancer, disease-modifying anti-rheumatic agents (DMARDs), direct acting antivirals, pulmonary arterial hypertension (PAH) and idiopathic pulmonary fibrosis (IPF), multiple sclerosis, specialty antipsychotics, and other medicines. These classes accounted for over 75% of spending in the specialty category and were defined to be mutually exclusive. Cancer claims were defined using the NDC codes for drugs used to treat cancer per prior work.[4] Direct acting antivirals claims were defined as those in ATC-Level 3 class J05A. Antipsychotic claims were defined as those in ATC-Level 3 class N05A.[iii] Multiple sclerosis[iv], PAH, and IPF medications[v] were defined based on NDCs for medicines approved for the treatment of these diseases by the Food and Drug Administration based on manual review. Finally, DMARDs were

---

[ii] Terms removed: phosphate, sodium, maleate, mesylate, tosylate, besylate, citrate, sulfate, oxalate, chloride, acid, calcium, succinate, tartrate, fumarate, acetate, carbonate, magnesium, dihydrate, benzoate, hydrochloride, and hcl, special characters "-" or ".", and the string "/pf" (referring to preservative free) when in the last position

[iii] Specialty anti-psychotic drugs with Part D claims in 2021 included: iloperidone, lurasidone, olzapine, paliperidone palmitate, pimavanserin, and risperidone microspheres

[iv] Specialty multiple sclerosis drugs with Part D claims in 2021 included: alemtuzumab, cladribine, fumarate, diroximel fumarate, fingolimod, glatiramer, interferon beta-1a, interferon beta-1b, monomethyl fumarate, natalizumab, ocrelizumab, ofatumumab, ozanimod, peg-interferon beta-1a, ponesimod, siponimod, teriflunomide

[v] Specialty PAH and IPF drugs with Part D claims in 2021 included: ambrisentan, bosentan, epoprostenol, macitentan, nintedanib, pirfenidone, riociguat, selexipag, and treprostinil

© 2025 Kakani P et al. *JAMA Health Forum.*

defined as NDCs for biologic products and JAK inhibitors approved for the treatment of auto-immune conditions including rheumatoid arthritis, ulcerative colitis, and psoriasis; conventional DMARDs (i.e., methotrexate, sulfasalazine, hydroxychloroquine, leflunomide, azathioprine, apremilast, cyclosporine, mycophenolate, and cyclophosphamide) were not included as they are virtually all non-specialty products.[vi] In four cases, where molecules were approved under different brand names or different formulations for multiple relevant conditions, the NDCs were assigned to the most appropriate category based on the specific brand name or formulation associated with the NDC.[vii] Two specialty NDC codes eligible for inclusion in multiple categories were assigned a single category based on the first approved indication by the FDA.[viii]

Non-specialty claims were stratified into select categories that together accounted for over 50% of spending for non-specialty drugs and were defined to be mutually exclusive. Specifically, we identified diabetes, anti-depressants, anti-anxiety, and statin medicines as those with NDCs associated with the ATC-Level 2 class A10, ATC-Level 3 class N06A, ATC-Level 3 class N05B, and ATC-Level 4 class C10AA respectively. Anti-hypertensives were defined as beta-blocking agents (ATC-Level 2 class C07), calcium channel blockers (ATC level 2 class C08), diuretics (ATC-Level 2 class C03), agents acting on the renin-angiotensin system (ATC level 2 class C09), and other antihypertensives (ATC-Level 2 class C02).[ix] Direct acting oral anti-coagulants billed in Part D in 2021 included apixaban, dabigatran, edoxaban, rivaroxaban.

eAppendix 4. Identifying pharmacy types

Pharmacies are frequently divided into classifications such as community pharmacy, specialty pharmacy, mail order pharmacy, etc. In the NCPDP data, pharmacies can self-report up to three pharmacy types. However, these data have numerous shortcomings. First, there is subjectivity in the interpretation of these categories. For example, specialty pharmacies are understood to focus on specialty drugs, but some large pharmacies that fill both specialty and non-specialty products list themselves as specialty pharmacies. Similarly, many pharmacies that consider themselves community pharmacies, such as many practice and health-system based pharmacies, actually dispense primarily specialty products. Second, we observed several important, high-spending pharmacies where self-reported pharmacy types appeared erroneous based on our manual review. For example, several large mail-order pharmacies were listed only as community pharmacies. These errors likely persist in part because pharmacies face no costs for incomplete reporting. Finally, given subjectivity in interpretation of these categories, many pharmacies do not report being the same type consistently over time.

Therefore, we used empirical patterns of drug dispensing to characterize pharmacy type. We distinguished between specialty and non-specialty pharmacies based on each pharmacy's share of Medicare Part D spending on specialty drugs. eFigure 1 depicts pharmacies ranked based on the share of Medicare Part D spending on specialty drugs on the x-axis and plots the cumulative share of drug spending on specialty drugs (in red) and non-specialty drugs (in blue) on the y-axis. Per Figure S1, 90.9% of spending on non-specialty products and only 10.3% of spending of specialty products occurs at pharmacies where less than 25% of spending is on specialty drugs. Thus, we defined specialty pharmacies as those where greater than 25% of spending is on specialty drugs and other pharmacies as non-specialty pharmacies. We could not empirically distinguish mail-order and other pharmacies, as information on whether prescriptions were mailed is not available in claims data.

The Insurer-PBM firms vary in the types of pharmacies each owns as outlined in eTable 2. For each insurer-PBM, eTable 2 presents the number of pharmacies and spending by pharmacies that are owned by CVS, UHG, Cigna, Humana, and Other Part D plans. The firms differ considerably in mean spending per pharmacy, reflecting differences in the types of pharmacies included and billing norms (e.g., the geographic breadth covered by each mail-order pharmacy). Thus, total spending by firm is not proportional to the number of pharmacies.

[vi] Specialty biologic & JAK inhibitor DMARDs with Part D claims in 2021 were: abatacept, adalimumab, anakinra, baricitinib, belimumab, canakinumab, certolizumab, etanercept, golimumab, guselkumab, infliximab (including biosimilars), ixekizumab, risankizumab, ruxolitinib, sarilumab, secukinumab, tocilizumab, tofacitinib, upadacitinib, ustekinumab, and vedolizumab.

[vii] NDCs for ruxolitinib were assigned to the DMARD category if the brand name was Opzelura and were assigned to the cancer category if the brand name was Jakafi; NDCs for ofatumumab were assigned to the multiple sclerosis category if the brand name was Kesimpta and to the cancer category if the brand name was Arzerra; NDCs for alemtuzumab were assigned to the multiple sclerosis category for the brand name Lemtrada and the cancer category for the brand name Campath; NDCs for cladribine were assigned to the multiple sclerosis category in the oral formulation and to the cancer category in the infused formulation.

[viii]Rituximab is a DMARD but was first approved for cancer and is thus assigned to the cancer class; mitoxantrone is approved for multiple sclerosis and cancer but was first approved for cancer and is thus assigned to the cancer class

[ix] To ensure categories were mutually exclusive, we excluded drugs approved for PAH and combination statin/anti-hypertensives from the anti-hypertensive category. Combination drugs including antipsychotics, antidepressants, and/or antianxiety medications were assigned to the antipsychotic category. Combination drugs including antidepressants and antianxiety medications were assigned to the antidepressant category.

© 2025 Kakani P et al. *JAMA Health Forum.*

eFigure 1. Cumulative share of spending on non-specialty and specialty drugs, by share of pharmacy spending on specialty drugs (2021)

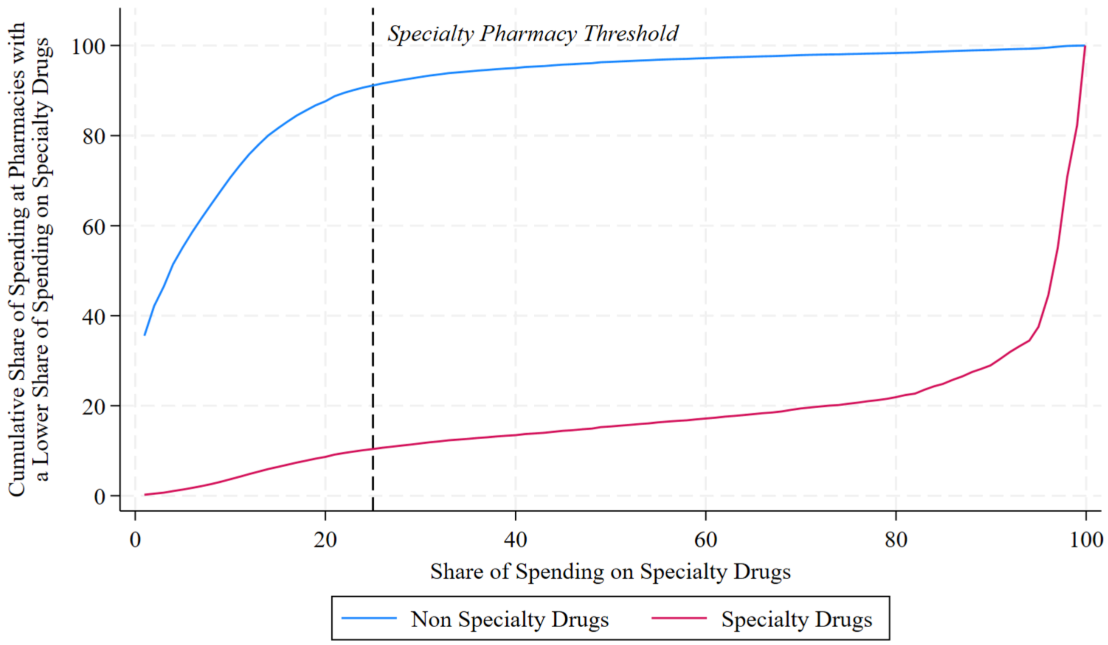

Note: The analysis is based on a 20% sample of Medicare Part D beneficiaries residing in the 50 United States and the District of Columbia in 2021 across all plans. Pharmacies are ranked based on their share of spending on specialty drugs on the x-axis. The y-axis illustrates the cumulative share of spending, among specialty and non-specialty drugs, accounted for by pharmacies with less than the share of spending on specialty drugs indicated on the x-axis.

eTable 2. Characteristics of pharmacies owned by CVS, UHG, Cigna, and Humana

	**Specialty pharmacies**			**Non-specialty pharmacies**			**Total**		
	Mean spending ($000s)	Total spending ($000s)	N pharmacies	Mean spending ($000s)	Total spending ($000s)	N pharmacies	Mean spending ($000s)	Total spending ($000s)	N pharmacies
CVS	5,996	2,392,663	399	581	5,706,985	9,828	792	8,099,648	10,227
UHG	3,079	1,841,321	598	16,893	1,199,427	71	4,545	3,040,748	669
Cigna	36,531	1,388,192	38	36,407	800,951	22	36,485	2,189,144	60
Humana	161,839	647,354	4	18,913	851,076	45	30,580	1,498,430	49
Other	1,314	10,616,877	8,082	371	18,063,708	48,717	505	28,680,586	56,799
**Total**	**1,851**	**16,886,408**	**9,121**	**454**	**26,622,147**	**58,683**	**642**	**43,508,556**	**67,804**

Note: The analysis is based on a 20% sample of Medicare Part D beneficiaries residing in the 50 United States and the District of Columbia in 2021 across all Part D plans.

© 2025 Kakani P et al. *JAMA Health Forum.*

eAppendix 5. Construction of market share terms underlying main text Figure 2 and Table

In Figure 2, we compared two quantities for the top-100 specialty and top-100 non-specialty molecules for each index insurer-PBM: (a) share of the index firm's insurer claims[x] filled by its owned pharmacies and (b) share of other firms' insurer claims filled by the index firm's owned pharmacies. We first compared these quantities, without considering geography where these two terms were formally defined as follows:

$$(a)\quad \alpha_{d,i}^{p} = \frac{\pi_{d,i}^{p}}{\pi_{d,i}} \qquad\qquad (b)\quad \beta_{d,i}^{p} = \frac{\pi_{d,-i}^{p}}{\pi_{d,-i}}$$

Here, $\alpha_{d,i}^{p}$ is the share of Part D insurer claims for molecule *d* paid by plans owned by index firm (*i*) that are filled through pharmacies (*p*) also owned by index firm *i*. Meanwhile, $\beta_{d,i}^{p}$ is the share of Part D insurer claims for molecule *d* paid by plans not owned by index firm *i* that are filled through pharmacies *(p)* owned index firm *i*. As an example, for index firm UHG and molecule imatinib, $\alpha_{d,i}^{p}$ would reflect the share of UHG Part D plan enrollees' imatinib prescriptions filled through pharmacies owned by UHG. Meanwhile, $\beta_{d,i}^{p}$ would reflect the imatinib prescriptions for Part D enrollees not in UHG plans that are filled through pharmacies owned by UHG. Mathematically, $\alpha_{d,i}^{p}$ and $\beta_{d,i}^{p}$ are constructed using the terms $\pi_{d,i}$, $\pi_{d,i}^{p}$, $\pi_{d,-i}$ and $\pi_{d,-i}^{p}$ defined as follows:

Variables

- $\pi_{d,i}$ = claims for molecule *d* paid by Part D plans owned by index firm *i*
- $\pi_{d,i}^{p}$ = claims for molecule *d* paid by Part D plans owned by index firm *i* and filled by pharmacies *(p)* owned by index firm *i.*
- $\pi_{d,-i}$ = claims for molecule *d* paid by Part D plans not owned by index firm *i*
- $\pi_{d,-i}^{p}$ = claims for molecule *d* paid by Part D plans not owned by index firm *i* and filled by pharmacies *(p)* owned by index firm *i.*

Figure 2 Panels A and B plot $\alpha_{d,i}^{p}$ (Y-axis) and $\beta_{d,i}^{p}$ (X-axis) for each molecule-index firm. Figure 2 Panels C and D report mean differences between $\alpha_{d,i}^{p}$ and $\beta_{d,i}^{p}$ as the main specification.

<u>Developing alternative geography-adjusted measures</u>

We developed alternative geography-adjusted measures of market share that equalize the distribution of insurers across counties. This analysis sought to address potential confounding that could, in theory, be caused by insurers operating in areas where their own pharmacies operate. For example, if CVS Part D plans are disproportionately located in areas with a high concentration of CVS pharmacy locations, then the analysis of steering that does not account for geography will find that enrollees in CVS plans disproportionately use CVS pharmacies. However, this would not be due to actions of plans to encourage use of pharmacies owned by the same parent firm.

To address this concern, we first estimated the previously identified quantities for each *molecule-index firm-county*, limiting to observations where the index firm's Part D plans and at least one other Part D plan fills 1 or more claims for the molecule. These exclusions are necessary to construct the measures and excluded a minority of claims (16.3% of specialty and < 1% of non-specialty index insurer claims respectively). We then aggregated each observation at the molecule-index firm level by weighting observations by the number of claims for the molecule filled by the index firm's Part D plans in the county. This retrieves for each index firm and molecule: (a) share of the index firm's insurer claims filled by its owned pharmacies, among counties where at least one other insurer also filled the molecule and (b) the share of the index firm's insurer claims that would have been filled by its owned pharmacies if the index insurer used their own pharmacies as much as other insurers located in the same counties. These terms adjusted for geography are expressed mathematically below:

[x] The term "index firm's insurer claims" refers to claims made by enrollees in Part D plans that are owned by the index firm. As noted in the limitations section, this analysis does not evaluate whether PBMs can steer patients to their own pharmacies, when the PBM is contracted by an external plan.

© 2025 Kakani P et al. *JAMA Health Forum.*

$$(a)\quad \theta_{d,i}^{p} = \frac{\sum_c \pi_{d,i,c}^{p}}{\sum_c \pi_{d,i,c}} \qquad\qquad (b)\ \gamma_{d,i}^{p} = \frac{\sum_c (\pi_{d,i,c}) * \frac{\pi_{d,-i,c}^{p}}{\pi_{d,-i,c}}}{\sum_c \pi_{d,i,c}}$$

In the above equations, $\theta_{d,i}^{p}$ is the share of claims for molecule *d* paid by Part D plans owned by index firm (*i*) filled through pharmacies (*p*) also owned by index firm *i*, among counties where at least one other Part D plan also had a claim for the molecule. $\gamma_{d,i}^{p}$ is the share of claims for molecule *d* paid by Part D plans owned by index firm (*i*) filled through pharmacies (*p*) also owned by index firm *i*, if the index firm's Part D plans used their own pharmacies as much as other plans located in the same counties. Mathematically, $\theta_{d,i}^{p}$ and $\gamma_{d,i}^{p}$ are constructed using the terms $\pi_{d,i,c}$, $\pi_{d,i,c}^{p}$, $\pi_{d,-i,c}$ and $\pi_{d,-i,c}^{p}$, defined as follows:

Variables

- $\pi_{d,i,c}$ = claims for molecule *d* paid by Part D plans owned by index firm *i* for patients living in county *c*
- $\pi_{d,i,c}^{p}$ = claims for molecule *d* paid by Part D plans owned by index firm *i* for patients living in county *c* and filled by pharmacies *(p)* owned by index firm *i.*
- $\pi_{d,-i,c}$ = claims for molecule *d* paid by Part D plans not owned by index firm *i* for patients living in county *c*
- $\pi_{d,-i,c}^{p}$ = claims for molecule *d* paid by Part D plans not owned by index firm *i* for patients living in county *c* and filled by pharmacies *(p)* owned by index firm *i.*

Sample Restrictions

- Restriction 1: $\pi_{d,-i,c} > 0$; this restriction limits to counties *c* where the molecule *d* is filled by at least one insurer besides the index insurer *i*
- Restriction 2: $\sum_c \pi_{d,i,c} > 0$; this restriction limits to *molecule-index insurer* pairs the molecule *d* is filled at least once by the index insurer in counties that satisfy restriction (1).

Notably, terms $\alpha_{d,i}^{p}$ and $\theta_{d,i}^{p}$ are equivalent except for the exclusion of certain counties *c*. Figure 2 Panels C and D report mean differences between $\theta_{d,i}^{p}$ and $\gamma_{d,i}^{p}$ as geography-adjusted estimates.

© 2025 Kakani P et al. *JAMA Health Forum.*

eAppendix 6. Supplemental analyses

eTable 3 provides the numerical estimates underlying Figure 1.

eTable 3. Share of Medicare Part D spending filled through pharmacies owned by CVS, UHG, Cigna, or Humana in 2021

	CVS	UHG	Cigna	Humana	Other
**Panel A. Overall and by pharmacy type**					
All	18.6%	7.0%	5.0%	3.4%	65.9%
Specialty pharmacies	21.4%	4.5%	3.0%	3.2%	67.9%
Non-specialty pharmacies	14.2%	10.9%	8.2%	3.8%	62.9%
**Panel B. Specialty drugs overall and by drug class**					
Overall	15.8%	10.7%	8.5%	4.0%	61.0%
Cancer	11.4%	13.0%	4.9%	3.2%	67.5%
DMARDs	17.4%	10.3%	7.5%	5.9%	58.9%
Anti-virals	14.1%	2.2%	0.8%	1.4%	81.5%
PAH & IPF	34.1%	5.9%	39.1%	10.6%	10.3%
Multiple sclerosis	31.2%	12.9%	9.6%	11.1%	35.2%
Anti-psychotics	13.2%	13.8%	1.3%	1.2%	70.5%
Other	14.4%	11.5%	12.7%	2.1%	59.3%
**Panel C. Non-specialty drugs overall and by drug class**					
Overall	20.2%	4.8%	3.0%	3.1%	68.8%
Diabetes	20.2%	4.6%	3.4%	4.1%	67.7%
NOAC	22.2%	5.3%	3.6%	3.9%	64.9%
Anti-hypertensives	21.6%	6.5%	3.8%	3.2%	64.9%
Anti-depressants	20.5%	6.0%	2.4%	2.0%	69.1%
Statins	23.3%	5.9%	4.6%	2.5%	63.7%
Anti-anxiety	21.1%	3.6%	1.3%	1.0%	73.1%
Other	19.4%	4.6%	2.4%	2.4%	71.3%

Note: The analysis is based on a 20% sample of Medicare Part D beneficiaries residing in the 50 United States and the District of Columbia in 2021 across all plans. Drug classes in Panels B and C are ordered from left to right by total spending, except for the "Other" category. Abbreviations include disease-modifying anti-rheumatic drugs (DMARD), pulmonary arterial hypertension (PAH), idiopathic pulmonary fibrosis (IPF), and UnitedHealth Group (UHG).

© 2025 Kakani P et al. *JAMA Health Forum.*

eTable 4 presents geography-adjusted estimated steering of claims for the top-100 specialty and top-100 non-specialty molecules by claim volume in 2021.

eTable 4. Geography-adjusted estimated steering of claims for top-100 specialty and non-specialty molecules to insurer-PBMs' owned pharmacies (2021)

	Share of the index firm's insurer claims filled by its owned pharmacies	Share of other firm's insurer claims filled by the index firm's owned pharmacies	Pair-wise difference		Sample (N)
	Median [IQR]	Median [IQR]	Median [IQR]	Mean (95% CI)	
**Panel A. Specialty Drugs**					
CVS	24.4 [16.5, 42.1]	13.1 [8.6,17.1]	11.8 [4.8, 26.8]	16.6 (13.8, 19.5)	100
UHC	18.5 [4.6, 30.9]	2.0 [0.6, 5.3]	9.2 [3.3, 26.9]	15.5 (12.3, 18.6)	100
Cigna	25.5 [5.0, 43.0]	1.6 [0.1, 4.6]	18.8 [4.4, 38.9]	22.5 (18.6, 26.4)	99
Humana	17.1 [5.5, 36.6]	0.1 [0.0, 0.5]	16.8 [5.4, 36.4]	21.8 (17.6, 26.0)	100
Across four firms	21.1 [6.6, 37.4]	1.8 [0.1, 8.5]	13.6 [4.0, 30.9]	19.1 (17.3, 20.9)	399
**Panel B. Non-specialty Drugs**					
CVS	30.4 [26.4, 34.5]	16.4 [15.4, 18.4]	12.9 [10.4, 15.7]	13.1 (12.0, 14.1)	100
UHC	11.8 [6.3, 17.3]	0.4 [0.3, 0.7]	11.2 [4.4, 16.5]	11.0 (9.6, 12.4)	100
Cigna	15.4 [5.7, 21.0]	0.4 [0.2, 0.6]	15.0 [5.6, 20.5]	14.1 (12.4, 15.8)	100
Humana	17.7 [9.0, 24.4]	0.0 [0.0, 0.0]	17.7 [9.0, 24.4]	16.8 (14.9, 18.6)	100
Across four firms	18.1 [8.9, 26.6]	0.4 [0.0, 9.1]	13.8 [7.3, 19.3]	13.7 (12.9, 14.5)	400

Note: The analysis is based on a 20% sample of Medicare Part D beneficiaries residing in the 50 United States and the District of Columbia in 2021 across all plans. Sample size for specialty drugs and Cigna was 99 because no Cigna beneficiaries had any prescriptions for 1 specialty molecule.

© 2025 Kakani P et al. *JAMA Health Forum*.

Finally, eFigure 2 illustrates an alternative measure of steering relative to the main text that aggregates across all claims for all drugs to provide a simpler set of summary statistics illustrating likely steering. For each index insurer-PBM firm the following two quantities: (a) the share of claims for the index firm's Part D plan enrollees filled at the index firm's pharmacies and (b) the share of claims for all other Part D plan enrollees filled at the index-firm's pharmacies. For example, in Panel A, the figure illustrates (a) the share of specialty drug claims by CVS Part D plan enrollees filled at CVS-owned pharmacies (26.4%) and (b) the share of spending drug claims by non-CVS Part D plan enrollees filled at CVS-owned pharmacies (12.8%). If insurer-PBMs did not steer and instead filled the same share of their spending at their own pharmacies as did other insurers, these quantities would be equivalent. However, unlike the main analysis, this analysis does not account for the theoretical possibility that insurer-PBM firms may specialize in serving patients using specific drug classes in both their plans and pharmacies, which could erroneously appear as steering; performing analysis at the drug-level addresses this concern and motivates the use of a drug-level analysis in the main text. Nonetheless, the differences between these two quantities are similar to the differences illustrated in the main text.

eFigure 2. Share of each insurer-PBMs' Part D plan claims filled through its own pharmacy versus the corresponding share among other Part D plans (2021)

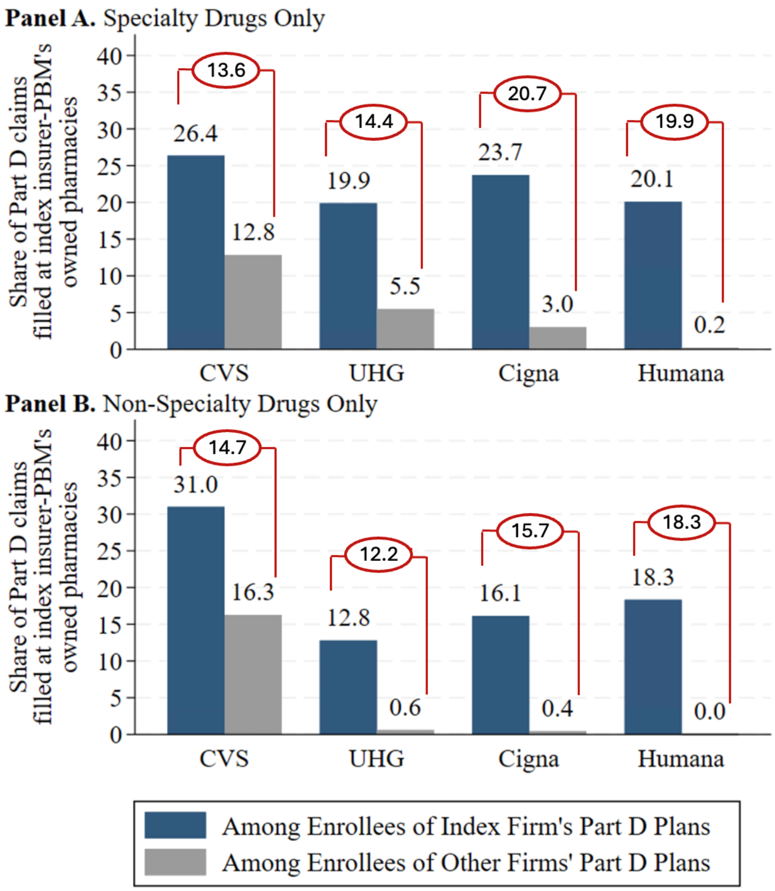

Note: The analysis is based on a 20% sample of Medicare Part D beneficiaries residing in the 50 United States and the District of Columbia in 2021 across all Part D plans. Each Panel of this figure illustrates the following two quantities for each index insurer-PBM firm: (a) the share of claims for the index firm's Part D plan enrollees filled at the index firm's pharmacies and (b) the share of claims for all other Part D plan enrollees filled at the index-firm's pharmacies. Panel A focuses on specialty drugs, and Panel B focuses on non-specialty drugs. For example, in Panel A, the figure illustrates (a) the share of specialty drug claims for CVS Part D plan enrollees filled at CVS-owned pharmacies (26.4%) and (b) the share of specialty drug claims by non-CVS Part D plan enrollees filled at CVS-owned pharmacies (12.8%). Differences between these two quantities are circled in red. Abbreviations include UnitedHealth Group (UHG).

© 2025 Kakani P et al. *JAMA Health Forum*.

eReferences

1. Kakani P, Cutler DM, Rosenthal MB, Keating NL. Trends in Integration Between Physician Organizations and Pharmacies for Self-Administered Drugs. *JAMA Network Open*. 2024;*7*(2):e2356592-e2356592.

2. Kanter GP, Parikh RB, Fisch MJ et al. Trends in medically integrated dispensing among oncology practices. *JCO Oncology Practice*. 2022;*18*(10):e1672-e1682.

3. Kaiser Family Foundation. Key Facts About Medicare Part D Enrollment, Premiums, and Cost Sharing in 2021. https://www.kff.org/medicare/issue-brief/key-facts-about-medicare-part-d-enrollment-premiums-and-cost-sharing-in-2021/ Accessed August 29, 2024.

4. Keating, NL, Jhatakia S, Brooks GA et al. Association of participation in the oncology care model with Medicare payments, utilization, care delivery, and quality outcomes. *JAMA*, 2021;*326*(18):1829-1839.

© 2025 Kakani P et al. *JAMA Health Forum*.
