# Supplementary material for: Use of and Steering to Pharmacies Owned by Insurers and Pharmacy Benefit Managers in Medicare
Source: JAMA Health Forum. 2025 Jan 10;6(1):e244874. doi: 10.1001/jamahealthforum.2024.4874 (PMC11724340; doi:10.1001/jamahealthforum.2024.4874)
Supplement: Supplement 2. — Data Sharing Statement [file jamahealthforum-e244874-s002.pdf]

Data Sharing Statement

Kakani. Use of and Steering to Pharmacies Owned by Insurers and Pharmacy Benefit Managers in Medicare. *JAMA Health Forum*. Published January 10, 2025. doi:10.1001/jamahealthforum.2024.4874

Data
Data available: No

Additional Information
Explanation for why data not available: The primary datasets used in this study are confidential datasets from the Centers for Medicare and Medicaid Services and the National Council for Prescription Drug Programs (NCPDP). These datasets were made available to us under data use agreements that do not permit sharing data with external entities. However, these datasets are available for request by other researchers.
